# Aberrant *SOX10* and *RET* expressions in patients with Hirschsprung disease

**DOI:** 10.1186/s12887-024-04682-6

**Published:** 2024-03-16

**Authors:** Gunadi, Verrell Christopher Amadeus, Fadila Dyah Trie Utami, Fiqih Vidiantoro Halim, Nabilah Anisa Novebri, Rahaditya Alrasyidi Hanggoro, Avinindita Nura Lestari, Kristy Iskandar, Andi Dwihantoro, Eko Purnomo

**Affiliations:** 1 Pediatric Surgery Division, Department of Surgery/Genetics Working Group/Translational Research Unit, Faculty of Medicine, Public Health and Nursing, Universitas Gadjah Mada/Dr. Sardjito Hospital, Jl. Kesehatan No. 1, Yogyakarta, 55281 Indonesia; 2 Department of Child Health/Genetics Working Group, Faculty of Medicine, Public Health and Nursing, Universitas Gadjah Mada/UGM Academic Hospital, Yogyakarta, 55291 Indonesia; 3 Pediatric Surgery Division, Department of Surgery, Faculty of Medicine, Public Health and Nursing, Universitas Gadjah Mada/Dr. Sardjito Hospital, Yogyakarta, 55281 Indonesia; 4 Pediatric Surgery Division, Department of Surgery/Genetics Working Group, Faculty of Medicine, Public Health and Nursing, Universitas Gadjah Mada/UGM Academic Hospital, Yogyakarta, 55281 Indonesia

**Keywords:** Hirschsprung disease, *SOX10*, *RET*, Enteric nervous system development, Gene regulatory network, Aberrant expressions

## Abstract

**Background:**

HSCR is a complex genetic disorder characterized by the absence of ganglion cells in the intestine, leading to a functional obstruction. It is due to a disruption of complex signaling pathways within the gene regulatory network (GRN) during the development of the enteric nervous system (ENS), including *SRY-Box Transcription Factor 10* (*SOX10*) and *REarranged during Transfection (RET)*. This study evaluated the expressions of *SOX10* and *RET* in HSCR patients in Indonesia.

**Methods:**

Total RNA of 19 HSCR ganglionic and aganglionic colons and 16 control colons were analyzed using quantitative real-time polymerase chain reaction for *SOX10* and *RET* with *GAPDH* as the reference gene. Livak’s method (2^−ΔΔC^_T_) was used to determine the expression levels of *SOX10* and *RET*.

**Results:**

Most patients were males (68.4%), in the short aganglionosis segment (78.9%), and had undergone transanal endorectal pull-through (36.6%). There were significant upregulated *SOX10* expressions in both ganglionic (2.84-fold) and aganglionic (3.72-fold) colon of HSCR patients compared to controls’ colon (ΔC_T_ 5.21 ± 2.04 vs. 6.71 ± 1.90; *p* = 0.032; and ΔC_T_ 4.82 ± 1.59 vs. 6.71 ± 1.90; *p* = 0.003; respectively). Interestingly, the *RET* expressions were significantly downregulated in both ganglionic (11.71-fold) and aganglionic (29.96-fold) colon of HSCR patients compared to controls’ colon (ΔC_T_ 12.54 ± 2.21 vs. 8.99 ± 3.13; *p* = 0.0004; and ΔC_T_ 13.90 ± 2.64 vs. 8.99 ± 3.13; *p* = 0.0001; respectively).

**Conclusions:**

Our study shows aberrant *SOX10* and *RET* expressions in HSCR patients, implying the critical role of *SOX10* and *RET* in the pathogenesis of HSCR, particularly in the Indonesian population. Our study further confirms the involvement of *SOX10-RET* within the GNR during the ENS development.

## Background

Hirschsprung disease (HSCR) is a complex congenital disorder characterized by the absence of intrinsic ganglion cells in the intestinal tract, starting distally and extending proximally to variable lengths [1–4]. Its incidence is higher in Indonesia (3.1:10,000) than in other populations, including Asians (2.8:10,000) and Caucasians (1.5:10,000) [1, 5]. These facts might be due to the greater risk allele frequency of *REarranged during Transfection (RET)* rs2506030 in Indonesia compared to other populations [6].

HSCR has been associated with more than 35 genes, including *SRY-box transcription factor 10 (SOX10)* and *RET* [1–5]. However, the thirty-five genes only provide 62% of the frequency of HSCR. Therefore, the pathogenesis of HSCR in the remaining patients remains unclear [2, 5]. HSCR can be associated with the alteration of gene expressions [7, 8].

*SOX10* is a transcription factor that affects *RET* expression by binding to one of its cis-regulatory elements (CREs) located in intron 1 of *RET: RET-7*, *RET-5.5*, and *RET + 3*. Therefore, in the presence of variants affecting CREs, *SOX10* is not able to bind anymore, leading to decreased *RET* expression [7]. Here, we aimed to evaluate the expressions of *SOX10* and *RET* in HSCR patients and compare them with the controls.

## Materials and methods

### Patients

Twenty patients under 18 years old presenting with non-syndromic HSCR whose ganglionic and aganglionic colons were collected during pull-through surgery are included in this study. We did not have any genetic data for those 20 HSCR patients. Sixteen control colon samples were collected during stoma closure for anorectal malformation patients. One HSCR patient was excluded due to low-quality RNA; thus, 19 of the total RNA of HSCR patients were further analyzed.

Each patient’s parents completed a written informed consent form before participating in this study. This study was approved by the Institutional Review Board of the Faculty of Medicine, Public Health, and Nursing, Universitas Gadjah Mada/Dr. Sardjito Hospital (KE/FK/0442/EC/2022 and KE/FK/0758/EC/2022). All experiments were conducted in conformity with the necessary legislation and guidelines.

### Total RNA isolation and quantitative real-time polymerase chain reaction

Total RNA extraction of the HSCR patient’s colon (aganglionic and ganglionic) and the control colon was done by using a total RNA Mini Kit (Tissue) (Geneaid Biotech Ltd., New Taipei City, Taiwan). The samples were stored at − 80 °C for future use.

One-step quantitative real-time polymerase chain reaction (qPCR) was performed using Kapa SBYR Fast qRT-PCR One Step Kit Universal (Kapa Biosystems, Massachusetts, USA) and BioRad CFX Real-Time PCR System (California, USA). The primers used for qPCR were as follows: *SOX10* 5’-ATGAACGCCTTCATGGTGTGGG-3’ (forward) and 5’-CGCTTGTCACTTTCGTTCAGCAG-3’ (reverse) [9], and *RET* 5′-CTGCCAAGTCCCGATG-3′ (forward) and 5′-TGGAGTACGCCAAATACG-3′ (reverse) [10]. The *GAPDH* gene was used as an internal control with the following primers: 5′-GCACCGTCAAGGCTGAGAAC-3′ (forward) and 5′-TGGTGAAGACGCCAGTGGA-3′ (reverse). As a reference gene, *GAPDH* has been validated in a diverse set of several tissues representing different organs, including the liver of biliary atresia and other diseases (gallbladder hydrops, omphalocele, internal bleeding, liver abscess, and choledochal cyst) and ganglionic and aganglionic colon of HSCR and anorectal malformation colon patients in our previous studies [11, 12]. The GAPDH showed a constant expression in a significant variation of human tissue samples [11, 12].

### Statistical analysis

The expression level of *SOX10* and *RET* was measured using the Livak method (2^− ΔΔC^_T_). The data is presented as mean ± SD and analyzed using an independent t-test; *p* < 0.05 was considered significant. All statistical analyses were performed using the IBM SPSS version 23 (Chicago, USA).

## Results

### Patient characteristics

Nineteen subjects were involved in this study. Most patients were males (68.4%) and short aganglionosis segment (78.9%) (Table 1).

**Table 1:** Characteristics of HSCR patients in this study

Characteristics	N (%), median (IQR)
Gender	
Male	13 (68.4)
Female	6 (31.6)
Age at pull-through (months)	18 (2.25–75)
Aganglionosis degree	
Short	15 (78.9)
Long	3 (15.8)
Total colon	1 (5.3)
Pull-through approach	
Transanal endorectal	7 (36.8)
Duhamel	6 (31.6)
Transanal Swenson-like	5 (26.3)
Transabdominal Soave	1 (5.3)

### SOX10 expressions in HSCR patients

**Fig. 1:**
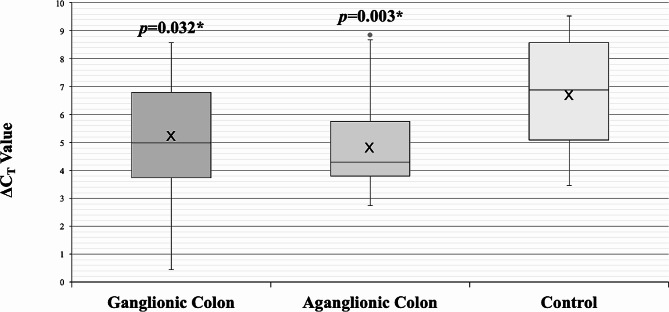
Box-plot graph of ΔC_T_ value of the *SOX10* expressions in HSCR ganglionic colon (ΔC_T_ 5.21 ± 2.04), HSCR aganglionic colon (ΔC_T_ 4.82 ± 1.59), and control colon (ΔC_T_ 6.71 ± 1.90). Box-plot graph of ΔC_T_ value reveals the median values as lines across the box. Lower and upper boxes represent the 25th percentile to the 75th percentile, while whiskers indicate the maximum and minimum values. *p < 0.05

There were significant upregulated *SOX10* expressions in both ganglionic (2.84-fold) and aganglionic (3.72-fold) colon of HSCR patients compared to controls’ colon (*p* = 0.032 and 0.003, respectively) (Table 2; Fig. 1).

**Table 2:** *SOX10* expressions in HSCR patients and controls

SOX10	ΔC_T_ ± SD	ΔΔC_T_ (95% CI)	Fold change	p-value
Ganglionic colon	5.21 ± 2.04	-1.50 (-2.87 – (-0.14))	2.84	0.032*
Aganglionic colon	4.82 ± 1.59	− 1.90 (-3.10 – (-0.69))	3.72	0.003*
Control colon	6.71 ± 1.90			

*p < 0.05 is considered statistically significant; CI, confidence interval; C_T_, cycle threshold; SD, standard deviation

### RET expressions in HSCR patients

**Fig. 2:**
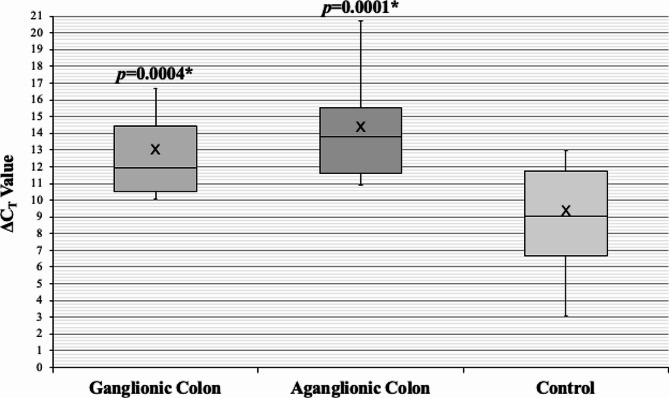
Box-plot graph of ΔC_T_ value of the *RET* expressions in HSCR ganglionic colon (ΔC_T_ 12.54 ± 2.21), HSCR aganglionic colon (ΔC_T_ 13.90 ± 2.64), and control colon (ΔC_T_ 8.99 ± 3.13). Box-plot graph of ΔC_T_ value reveals the median values as lines across the box. Lower and upper boxes represent the 25th percentile to the 75th percentile, while whiskers indicate the maximum and minimum values. *p < 0.05

Subsequently, we determined the *RET* expressions in HSCR patients and controls. Interestingly, significant downregulated *RET* expressions were noted in both ganglionic (11.71-fold) and aganglionic (29.96-fold) colon of HSCR patients compared to controls’ colon (*p* = 0.0004 and 0.0001, respectively) (Table 3; Fig. 2).

**Table 3:** *RET* expressions in HSCR patients and controls

RET	ΔC_T_ ± SD	ΔΔC_T_ (95% CI)	Fold change	p-value
Ganglionic colon	12.54 ± 2.21	3.55 (1.71–5.39)	11.71	0.0004*
Aganglionic colon	13.90 ± 2.64	4.91 (3.01–6.80)	29.96	0.0001*
Control colon	8.99 ± 3.13			

*p < 0.05 is considered statistically significant; CI, confidence interval; C_T_, cycle threshold; SD, standard deviation

## Discussion

Our study is able to show the aberrant *SOX10* and *RET* expressions in HSCR patients. A previous study showed lower *SOX10* expressions were associated with hypertrophic nerve trunks in HSCR patients [8]. They suggested that the aberrant *SOX10* expressions might involve HSCR pathogenesis via interaction with other neurotrophic factors in non-syndromic patients without any pathogenic variants in the *SOX10* gene. Our study provides new evidence of the aberrant *SOX10* expressions in HSCR patients from a different population from a previous study [9]. In addition, most *SOX10* pathogenic variants were found in syndromic HSCR patients [13]. Interestingly, a recent study revealed that three common variants within CREs of *RET* decreased the binding of transcription factors, including *SOX10*, to those three CREs. These interactions caused the decrease of *RET* expressions and disruption of other HSCR and enteric nervous system (ENS) genes within the *RET–EDNRB* GRN [7].

*SOX10* is a gene encoding a member of the SRY-related HMG-box (SOX) family of transcription factors that regulate embryonic development and determine cell fate. The SOX10 protein acts as a nucleocytoplasmic shuttle protein, essential for neurogenesis and neural crest cells (NCCs) development [13]. *SOX10* has significant roles in the development of NCCs, one of which is regulating the migration of NCCs, which form the ganglionic plexus of the ENS [14]. *SOX10* helps ensure the survival and pluripotency of NCCs during and after migration and contributes to determining their fates and differentiation [14–16]. It is also known that direct *SOX10* interaction with *Cadherin-19* (*Cdh19*) mediates early sacral NCCs migration by forming cadherin-catenin complexes. These complexes interact with the cytoskeleton filamentous actin in the migration of NCCs [17].

*SOX10* expression is regulated by several transcription factors, such as *SOX9, Olig2, WNT, FoxD3*, and Snail [16, 18]. Overexpression of *SOX10* has been shown to inhibit the differentiation of NCCs [14, 18]. After the differentiation of the NCCs, *SOX10* expression is maintained in enteric glial cells while downregulated in neurons and smooth muscle cells [14, 16].

Several weaknesses of our study were noted, including a small sample size, and our findings did not consider other ENS and HSCR gene expressions involved within the *RET–EDNRB* gene regulatory network (GRN). Two housekeeping genes should always be included to account for technical variations during qPCR. Our study only used *GAPDH* as an internal control. It is essential to conduct a further study to determine whether the increased *SOX10* expressions are due to the increased glial cells or enhancement of *SOX10* expressions in ENS cells. Moreover, it is also interesting to determine whether the increased *SOX10* expressions due to the cell numbers of *SOX10-*expressing cells are changed or the *SOX10* promoter activity is enhanced. In the postnatal period, *SOX10* is mainly expressed in glial cells. The quantification of the glial cell population in the colon from patients and control is crucial. Checking aberrant expression of *SOX10* in other cell types is also needed. Alternatively, an isolated explant or cell culture experiment is required. Using this system, it is possible to estimate promoter or enhancer activities of *SOX10* and high levels of *SOX10* in ENS cells. In addition, we do not validate the protein levels of SOX10, including immunohistochemistry, in HSCR patients due to limited resources. Pathogenic variants in *GLI*, resulting in upregulated *Sox10* expression in vitro, have been detected in patients with non-syndromic HSCR [19]. Therefore, screening pathogenic variants in transcription factors regulating *SOX10* in patients or estimating promoter or enhancer activities of *SOX10* in isolated human ENS cells is essential.

*SOX10* is required for *RET* expression, and decreased *RET* expression causes HSCR [7, 14, 20]. During the development of ENS, *SOX10* controls specific genes, including *RET, EDNRB*, and *SOX10* itself [14]. The decrease of *RET* expressions disrupts the other HSCR and enteric nervous system (ENS) genes within the *RET–EDNRB* GRN, including *GATA2, SOX10, RARB*, and *NKX2.5* [21]. However, no direct evidence indicates that increased *SOX10* leads to aganglionosis, i.e., HSCR. Interestingly, upregulated *SOX10* expression promotes the migration of neural crest-like cells of the neural tube; however, it hampers their differentiation [22]. We further determined the *RET* expressions in our HSCR patients. Intriguingly, the expressions of *RET* were significantly downregulated in patients compared to controls. These decreased *RET* expressions might lead to HSCR. It is important and interesting to conduct a further study on how upregulated *SOX10* causes decreased *RET* expression, resulting in HSCR.

Moreover, our findings might be beneficial during the surgical counseling to the parents that in a polygenic disorder, such as HSCR, a complex interaction between genes might result in different disease phenotypes. This evidence further confirms the complexity of the pathogenesis of HSCR, including the disruption of the GNR during the ENS development.

## Conclusions

Our study shows aberrant *SOX10* and *RET* expressions in HSCR patients, implying the critical role of *SOX10* and *RET* in the pathogenesis of HSCR, particularly in the Indonesian population. Our study further confirms the involvement of *SOX10-RET* within the GNR during the ENS development.

## Data Availability

All data generated or analyzed during this study are included in the submission. The raw data are available from the corresponding author upon reasonable request.

## Abbreviations

*SOX10*: *SRY-Box Transcription Factor 10*
*RET*: *REarranged during Transfection*
*GAPDH*: *glyceraldehyde-3-phosphate dehydrogenase*
WS4: Waardenburg syndrome type 4, HSCR:Hirschsprung disease
qPCR: quantitative real-time polymerase chain reaction
